# Trace Element Uptake by Willows Used for the Phytoremediation of Biosolids

**DOI:** 10.3390/life13010243

**Published:** 2023-01-16

**Authors:** Angela Contangelo, Juergen Esperschuetz, Brett H. Robinson

**Affiliations:** 1 School of Agricultural Science, Forestry, Food and Environmental Sciences, University of Basilicata, 75100 Matera, Italy; 2 School of Forestry, Faculty of Engineering, University of Canterbury, Ilam Campus, Christchurch 8041, New Zealand; 3 School of Physical and Chemical Sciences, Faculty of Science, University of Canterbury, Ilam Campus, Christchurch 8041, New Zealand

**Keywords:** biochar, sewage sludge, phytomanagement, cadmium, copper

## Abstract

The land application of biosolids can result in the unacceptable accumulation of Trace Elements (TEs) in agricultural soil and potentially introduce xenobiotics and pathogens into the food chain. Phytoremediation of biosolids aims to minimize this risk, while producing valuable biomass. Willows, well known to accumulate zinc (Zn), are used extensively in farming systems for soil conservation, shelter and as feed supplements with demonstrable health benefits. Potentially, biosolids phytoremediation could occur on marginal lands adjacent to farmlands where willows are grown for supplementary fodder. We aimed to determine the uptake and distribution of Zn and other TEs in willows grown on soils amended with biosolids and biosolids blended with biochar, with a view to their use as stock fodder. In the Canterbury Region, New Zealand, we grew *Salix* ‘tangaio’ (*S. matsudana* X *S. alba*) in a greenhouse trial and field study. The biomass production of the willows was unaffected by biosolids and increased by the biosolids+biochar mixture. The addition of 4% biosolids (*w*/*w*) to the soil resulted in a foliar Zn concentration of 600–1000 mg kg^−1^, some 25 times higher than the average New Zealand pasture. Zinc concentrations were highest in the bottom leaves and increased throughout the season. Biosolids addition doubled the copper (Cu) concentration to 10 mg kg^−1^. Adding biochar to the system reduced the plant uptake of Cu and to a lesser extent Zn, while cadmium (Cd) uptake was unaffected. For Cd, Cu, and Zn, plant uptake was a function of the Ca(NO_3_)_2_-extractable concentration, both in greenhouse experiments and the field trial. Future work should determine the changes in plant TE uptake over several growing seasons.

## 1. Introduction

Biosolids, a solid fraction of sewage treatment, are rich in organic matter and plant nutrients, however, they may also contain elevated concentrations of contaminants, especially, xenobiotics, pathogens, and non-essential Trace Elements (TEs) [1,2,3]. Biosolids are commonly applied to land as a soil conditioner [4]; however, application to agricultural soil can endanger food safety [5] and result in unacceptable accumulation of phosphorus (P) and TEs [6]. However, TE deficiencies are widespread in agricultural systems, reducing both productivity and food quality [7,8]. Many soils are deficient in Cu and Zn, and marginal deficiency in sheep and cattle is associated with reduced growth and fecundity [9,10]. Supplements of Zn are used to protect both sheep and cattle against fungal-derived facial eczema [8].

Phytoremediation/phytomanagement of biosolids could occur on marginal lands, where a limited number of biosolids applications are used to establish plants that provide economic or environmental value. Part of this value may come from increased concentrations of essential TEs in the plants that are subsequently fed to livestock. On acidic soils with low concentrations of trace elements, the addition of biosolids can increase plant uptake of Zn, but also the non-essential and toxic Cd [11]. Nevertheless, biosolids add adsorptive phases to soils [12] that reduce TE availability to plants [13]. Stadelmann and Furrer [14] reported that the concomitant effect of increased organic matter in the soil increased soil CEC from 17.2 cmol_c_ kg^−1^ to 23.7 cmol_c_ kg^−1^ from a biosolids application rate of 5 t/ha over 7 years. In biosolids, Zn is strongly bound to the solid organic matrix, and the addition of biosolids to soil with high pH and high Zn concentration may immobilize soil-borne Zn, thereby reducing plant uptake [15]. Increasing soil concentrations of lead (Pb), chromium (Cr), and Cu may not lead to increases in plant uptake because these elements are immobilized in plant roots [16] as they bind to the root cortex and have limited transport across the endodermis into the root xylem.

The rates of biosolids addition required to significantly increase essential micronutrient concentrations in plants [11] may result in excessive nitrate leaching into ground and surface waters [17]. Potentially, high rates of biosolids could be blended with other carbon-rich materials, such as biochar, to offset nitrate leaching [18,19]. Biochar is produced by pyrolyzing biomass in a low-oxygen environment [20]. A wide variety of organic materials are available for its production, including forestry and crop residues, paper mill sludge, and poultry waste [21]. Biochar can improve soil water retention [22,23], reduce acidity [24], increase cation exchange capacity [25], increase nitrogen (N) retention [26,27], mitigate N_2_O emissions [28], increase microbial activity [29], and strengthen mycorrhizal associations [30,31]. Due to negatively charged organic functional groups, biochar can form complexes with TE cations, thereby reducing their mobility in soils and waters [32,33]. This may lead to a reduction in plant uptake of these elements [33], thereby offsetting the potential biofortification benefits of adding biosolids. The influence of biochar on soil properties is likely to vary significantly depending on the original material, since biochar properties are governed by the biomass source and the pyrolysis conditions [34,35,36].

Poplars (*Populus* spp.) and willows (*Salix* spp.) are commonly used in agricultural systems because of their rapid growth, high transpiration rate, extensive root systems, and palatability to stock [37,38]. These trees are extensively grown for soil conservation, since they can intercept runoff and reduce N leaching, but also provide nutrient-rich stock fodder in times of drought [39,40]. Both foliage and small twigs can be used as a valuable source of fodder to sustain live weight gain [38,41], and in addition provide an emergency food source with proven health benefits [42]. Both poplars and willows have been shown to accumulate higher concentrations of Zn, Cd, and boron (B) relative to pasture species [43,44,45]. Willows are known as leaf accumulators for Zn and Cd and root accumulators for Cu, Cr, nickel (Ni), and Pb [46,47,48]. Trace element accumulation by willows is dependent on species [49,50,51], clone [51,52], growth performance [53], root distribution [54], and sampling period [50,51].

We hypothesized that on unfertilized marginal farmland, biosolids would significantly increase the concentrations of Zn and Cd in the above-ground portions of willow and that this increase will be offset by blending biosolids with biochar. Further, we hypothesized that there will be only small increases in foliar Cu, Pb, and other trace elements on biosolids-amended soils. We aimed to determine whether phytoremediation using willows amended with biosolids and biosolids/biochar mixtures could be used to produce biomass that was biofortified in essential trace elements, while maintaining non-essential TEs below critical thresholds.

## 2. Materials and Methods

### 2.1. Greenhouse Experiment 

We collected 500 kg of Templeton Fine Sandy loam (an Immature Pallic soil) from the Lincoln University Dairy Farm (Lincoln, New Zealand, 43°38′12″ S, 172°26′17″ E). Following removal of the surface litter and vegetation, soil was collected from the top 0.2 m, representing the ‘A’ horizon. Biosolids (160 kg) were obtained from the Kaikoura regional treatment works, Kaikoura, New Zealand 42°21′38″ S, 173°41′25″ E). The biosolids were acidic (Table S1) and had elevated concentrations of plant nutrients and some TEs. Soils and biosolids were homogenized using a concrete mixer and passed through a 20 mm sieve. The biochar was manufactured from Pinus radiata D. Don as described in Clough et al. [55] and Taghizadeh-Toosi et al. [56]. The biochar was crushed into particles with a maximum size of 10 mm. The chemical properties of the soils, biosolids, and biochar used in these experiments is given in Table S1. 

Four treatments (control, “biosolids”, “biochar”, “biosolids/biochar”) were replicated five times. For each treatment, biosolids, biochar, or a mixture of two were mixed into the soil at a rate of 4% (*w*/*w*). On the 15th of September 2010, the treatments were filled into 15 L pots in a greenhouse at Lincoln University (Lincoln, New Zealand). The pots were left for three weeks to equilibrate. Subsamples were taken from each pot at a depth of 0–0.1 m. On the 8th of October 2010, one *Salix* “tangoio” (*S. matsudana* X *S. alba*) seedling with an aboveground biomass dry weight of 75 g was planted into each container. The pots were arranged in a randomized block design. During the experimental period of three months, all treatments were maintained at field water capacity. Temperatures ranged between 9 °C and 16 °C during the nighttime (10 pm until 6 am) and between 14 °C and 28 °C during the daytime.

Leaves were sampled monthly until the final harvest on the 6 January 2011. At the end of the experiment, a destructive harvest was carried out. Aboveground plant material was isolated from roots, and further separated into trunks, stems and leaves, whereas the trunk was subdivided into basal, median, and apical material. All plant parts were thoroughly washed with deionized water and oven-dried in paper bags at 105 °C until constant weight was obtained. The dry biomass was recorded prior to grinding (Ika Yellowline Analytical Grinder A10) and storage for analysis.

### 2.2. Field Experiment

A field trial was carried out contemporaneously to compare with the greenhouse experiment. The field trial occurred at the Lincoln University Dairy Farm (Lincoln, New Zealand; 43°38′12″ S, 172°26′17″ E). The soil type (Templeton Fine Sandy Loam) was the same as that used for the pot trial, with the soil properties shown in (Table 1). The experiment was carried out on six 10 m × 5 m plots. Four plots were amended with biosolids (applied at a rate of 400 kg N ha^−1^ equiv.) and spiked with increasing concentrations of Zn to give soil concentrations of 50, 100, 150, and 200 mg kg^−1^. A fifth “unspiked” plot was amended with only biosolids at the same rate of 400 kg N ha^−1^ equiv., and the sixth plot has not received any biosolids application and was treated as a “control” plot. 

For the field experiment, biosolids were obtained from the Christchurch City sewage treatment works at Bromley (Christchurch, New Zealand) and incubated in polypropylene tanks prior to application. For each biosolids treatment, approximately 250 L of anaerobically digested sewage biosolids (8% solids) were placed in a tank and spiked with Zn concentrations (ZnSO_4_ × 7H_2_O) according to the treatments above. Following Zn addition, the biosolids mixtures were thoroughly stirred and the tanks tightly sealed. Biosolids for the “unspiked” treatment were incubated in a separate tank without receiving additional TEs. The samples were held in this condition for six months at ambient temperature with occasional stirring. Although stirring would have introduced some air into the biosolids, the thick consistency, small surface area to volume ratio, and the tight sealing of the tank lids ensured that the samples remained in a predominantly anaerobic condition during incubation. After the six months of incubation, the tanks were transported to the field site. Biosolids were applied to the soil surface with subsequent rotavation within the top 0.1 m. In July 2010, each plot was planted with 24 one-year-old willows (“tangaio”) with a distance of 1 m × 1 m between plants, leaving 1.5 m buffer strips. Mean daily temperature increased from 6.3 °C in July to 17.1 °C in January. Rainfall during the experimental period (ca. 400 mm) was supplemented with irrigation in October–January to ensure the plants did not suffer from water stress. On the 6 January 2011, a representative leaf sample, including basal, media, and apical leaves, was taken from five trees from each plot. Only internal (non-edge) trees were sampled. 

### 2.3. Chemical Analysis

A representative sample of 0.5 g of ground material was dissolved in 8 mL of AristarTM nitric acid (HNO_3_, ±69%). The digestion was carried out in Teflon vessels using CEM MARSXpress microwave digestion system, of which parameters were set to 1600 W, ramping at 170 °C in 20 min, and to holding 170 °C for 20 min. After cooling, the vessel contents were filtered using Whatman^TM^ N°52 filter paper and diluted with milliQ water to a volume of 25 mL prior to analysis.

Soil samples were oven-dried at 105 °C in paper bags until constant weight and subsequently sieved through a Nylon sieve (≤2 mm). Soil pH was determined in water (2.5:1, water: soil) using a Mettler Toledo pH meter. To determine the trace elements in the soil solution, 30 mL of 0.05 M Ca(NO_3_)_2_ was added to 5 g ± 0.05 g dried and sieved soil and subsequently end-over-end shaken for 2 h. The samples were then centrifuged and filtered using Whatman^TM^ N°52 filter paper [57].

Following the method of Black et al. [58], we carried out extractions using 0.05 M Ca(NO_3_)_2_ to estimate the concentrations of elements in soil solution. Soil samples (5 g) were accurately weighed into 50 mL centrifuge tubes. Into each tube, 30 mL of solution were added, and the mixture was agitated for 2 h on an end-over-end shaker. Mixtures were filtered with Whatman 52 filter paper and stored at −18 °C for chemical analysis.

Digests and extracts were analyzed by Inductively Coupled Plasma Optical Emission Spectrometry (ICP-OES) (Varian 720 ES). Blanks were included within each digestion batch to allow corrections for any contamination from chemicals during the digestion process. Standard Reference Materials, (SRM 1573a and Wageningen SRM IPE100) were analyzed for quality assurance purposes alongside the samples. Results of the SRMs were within 92–109% of the certified values. Elemental analyses for C and N concentrations were carried out using an Elementar Vario MAX CN analyzer.

### 2.4. Statistical Analysis

All data were analyzed by PASW Statistic 17.0. After ensuring a homogeneity of variances using a Levene test, normal distribution of the data was verified by a Shapiro–Wilk test. ANOVA analysis has been performed to test the null hypothesis that the means of all the groups being compared are equal. An LSD post-hoc test has been used to identify significant differences *p* ≤ 0.05 between means where the null hypothesis has been rejected. When Levene statistic failed and ANOVA had rejected the null hypothesis, the Welch and Brown–Forsythe statistics (robust test of equality of means) was run to compare means, followed by the Games–Howell test, a post-hoc test that does not rely on homogeneity of variance. 

## 3. Results

**Figure 1:**
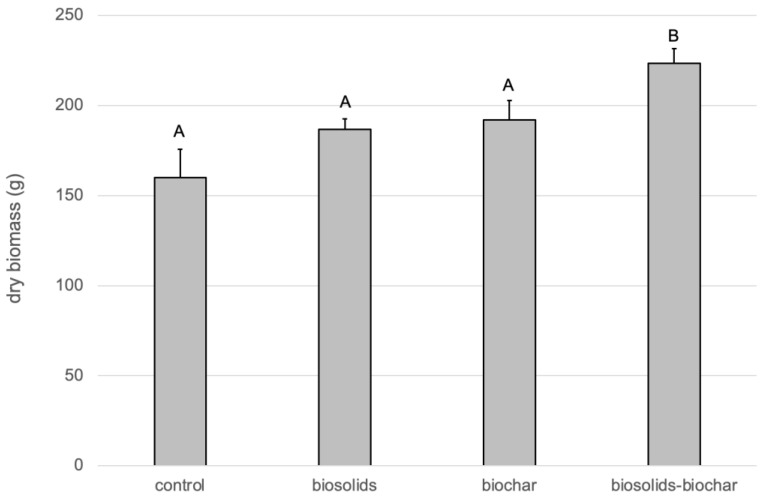
The total above-ground biomass (g dry matter) produced during the pot trial. Bars with the same letter are not significantly different. The error bars represent the standard error of the mean (n = 5).

The addition of biosolids increased the concentrations of carbon and plant macronutrients (N, P, K, S) in the substrates (Table 1), since they contained significantly higher concentrations of these elements than the control soil (Table S1). Biochar increased the carbon content of the soil and gave lesser increases in plant nutrients. At the final harvest, the “biosolids/biochar” treatment had significantly higher total biomass (Figure 1) compared to “biosolids”, “biochar”, and “control” treatments, which were not significantly different. The results show that neither biosolids nor biochar treatments in isolation resulted in a significant biomass increase but a combination of the two increased the average biomass by 40%.

**Figure 2:**
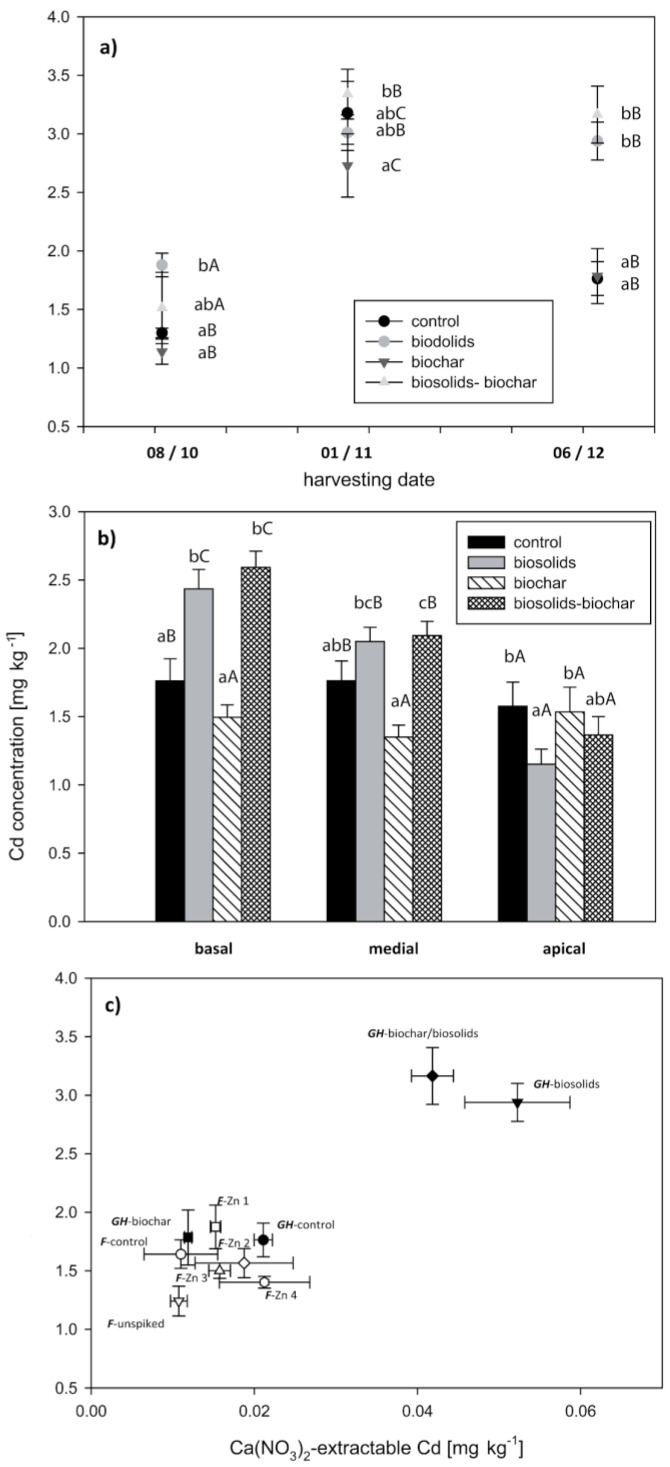
Foliar Cd concentrations as a function of harvest date (**a**), leaf position (**b**), and Ca(NO_3_)_2_—extractable Cd in soil in both greenhouse (GH) and field (F) grown willows (**c**). Error bars represent the standard error of the mean (n = 5). Bars with the same letter are not significantly different. Lowercase letters represent significance between treatments. Uppercase letters represent significance between harvest date (**a**) or leaf position (**b**).

**Figure 3:**
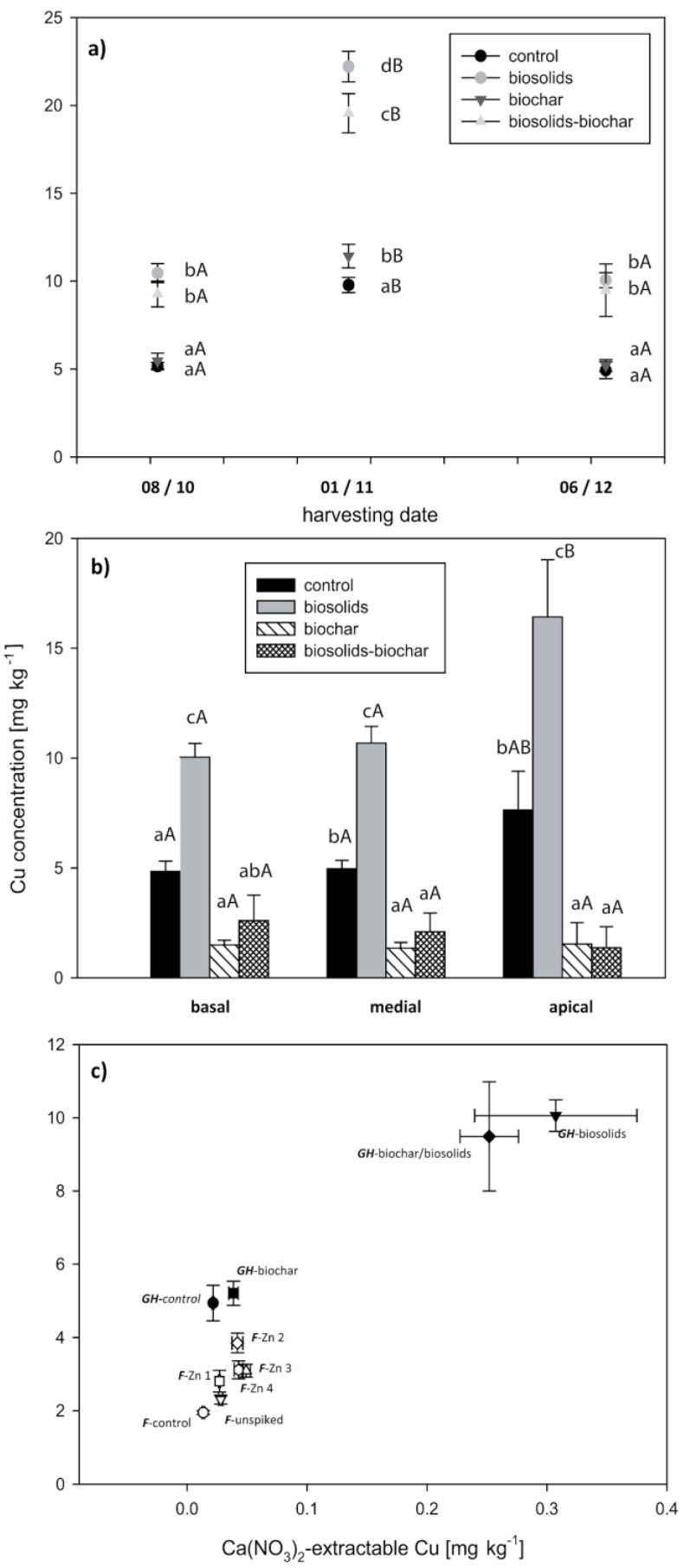
Foliar Cu concentrations as a function of harvest date (**a**), leaf position (**b**), and Ca(NO_3_)_2_—extractable Cu in soil in both greenhouse (GH) and field (F) grown willows (**c**). Error bars represent the standard error of the mean (n = 5). Bars with the same letter are not significantly different. Lowercase letters represent significance between treatments. Uppercase letters represent significance between harvest date (**a**) or leaf position (**b**).

**Figure 4:**
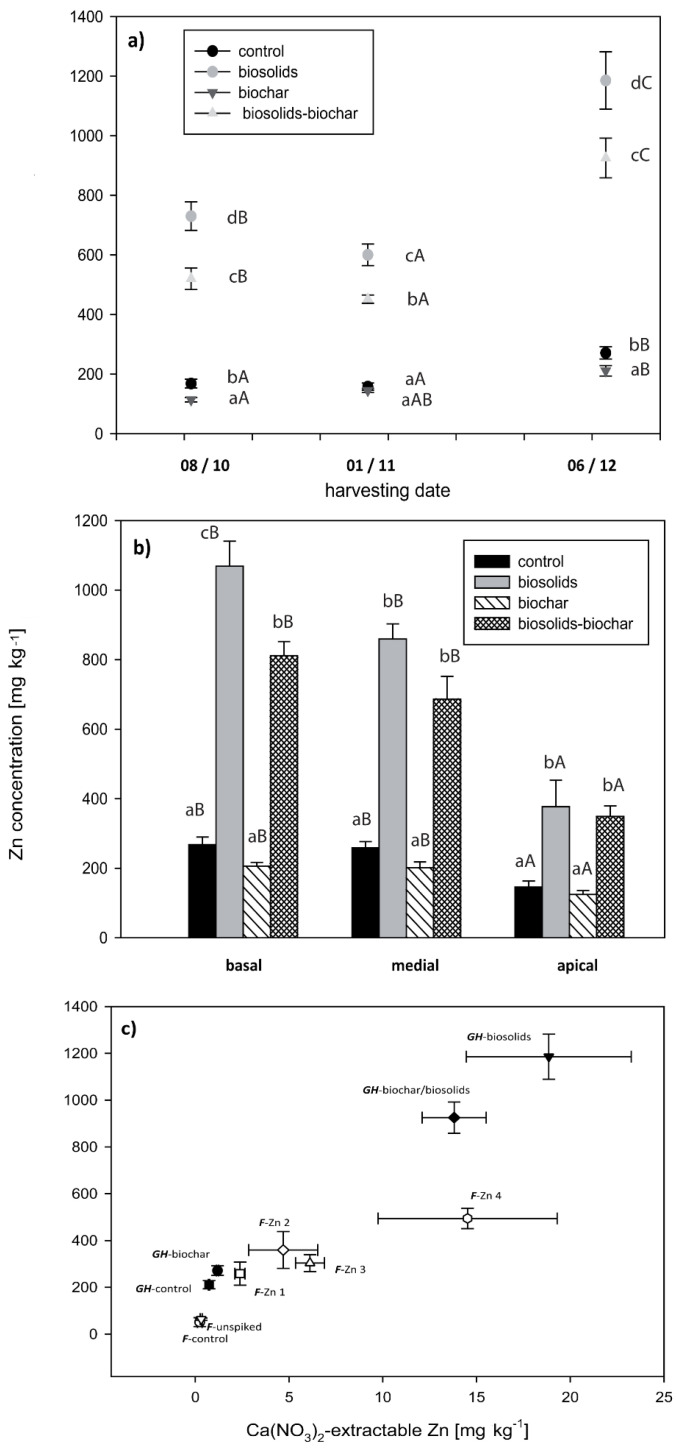
Foliar Zn concentrations as a function of harvest date (**a**), leaf position (**b**), and Ca(NO_3_)_2_—extractable Zn in soil in both greenhouse (GH) and field (F) grown willows (**c**). Error bars represent the standard error of the mean (n = 5). Bars with the same letter are not significantly different. Lowercase letters represent significance between treatments. Uppercase letters represent significance between harvest date (**a**) or leaf position (**b**).

The addition of biosolids significantly increased the total concentrations of Cd, Cu, and Zn (by factors of 1.3, 3.5, and 1.5, respectively), while there were only small differences in the other TEs. The increase in soluble concentrations of these TEs was even greater (by factors of 2.5, >10, and 9.4, respectively). These increases were reflected in significantly higher concentrations of these elements in the plant leaf tissue (Figure 2, Figure 3 and Figure 4). For Cu (Figure 3a,b), and to a lesser extent Zn (Figure 4a,b), the increase in concentration following biosolids addition was offset by the addition of biochar. Biochar had little effect on plant Cd-uptake (Figure 2a,b).

Within treatments, the Cd and Zn concentrations increased over time (Figure 2a and Figure 4a, respectively). There was no consistent temporal trend for Cu (Figure 3a). After three months of growth, there were significant differences in the trace element concentrations between treatments. In all treatments, the basal leaves had significantly higher Cd and Zn concentrations than the medial and apical leaves (Figure 2b and Figure 4b). This difference was greatest in the “biosolids” and “biosolids/biochar” treatments. In contrast to Cd and Zn, the apical leaves had the highest Cu concentrations in the treatments that included biosolids (Figure 3b). In both the pot trial and the greenhouse trial, the foliar TE concentration was significantly positively correlated with the Ca(NO_3_)_2_-extractable TE (Figure 2c, Figure 3c, and Figure 4c), whereas the correlation with the total TE concentration was weaker. At the final harvest, the lower foliar bioaccumulation coefficients (plant/soil concentration quotients) in the biosolids treatments for Cd, Cu, and Zn were 3.5–6.5, 0.1–0.3, and 4.0–10.0, respectively. Concentrations of Cu and Zn in the shoots were significantly lower than in the leaves, typically 10–30% of the foliar concentration (Tables S7–S9); however, Cd concentrations in the shoots were similar to the foliar concentrations.

## 4. Discussion

Biosolids increased the growth of willows, but only when it was combined with biochar. In acid soils, other authors [11,59] have reported similar findings. The effect may be due to a pH increase following biochar addition. The biosolids used in these experiments were acidic (pH 4.1, Table S1) and the nutrients contained therein may not have been bioavailable. When combined with alkali biochar (pH 7.8 Table S1), the liming effect [60] may increase the bioavailability of N and other plant macronutrients in the soil. Without biosolids, biochar had no effect on plant growth. The results indicate that biosolids can accelerate willow growth provided pH is controlled. On non-acid soils, liming agents such as biochar may not be required.

Cadmium concentrations were below the threshold of 5 mg kg^−1^, above which animal health may be affected [61]. However, on biosolids-amended soil, Cd concentrations were significantly higher than the threshold of 1 mg kg^−1^ set by the European Union as a safety threshold for animal fodder [62]. Given that willows would only form a small part of the animals’ diets, it is unlikely that using biosolids-grown willow as supplementary fodder would increase the average Cd concentration of the whole diet above 1 mg kg^−1^. Moreover, the bioaccessibility and toxicity of the Cd in the willow leaves may be reduced by the elevated Zn concentrations [63,64]. Nevertheless, the effects of Cd on animal production and food quality should be determined before the large-scale phytoremediation of biosolids using willows is implemented.

Copper concentrations in biosolids-amended soils were significantly higher than the controls. Similar results, both in terms of total concentration and response to biosolids have been shown for *Lolium multiflorum* L. [65]. This increase is significant because Cu-deficiency is widespread in agricultural systems. For example, some 25% of New Zealand soils contain insufficient Cu for animal agriculture [66]. As with Cd, there is a lacuna of information on the bioaccessibility of Cu in willow leaves used for stock fodder.

When grown in biosolids-amended soil, willows accumulated agronomically significant concentrations of Zn that may provide a prophylaxis against facial eczema [8] or alleviate Zn deficiency, which is widespread in many agricultural systems [66,67]. The Zn contained in willow foliage has been demonstrated to be bioaccessible to sheep [8] and therefore likely bioaccessible to other ruminant animals. The increase in Zn in willows is consistent with increases in other plant species following biosolids addition [11,65,68], although the magnitude of the increase was significantly greater in this study. This may have been due to increased Zn bioavailability due to the low soil pH (Table S1) or due to the propensity of willows to accumulate inordinate concentrations of Zn [69]. At higher soil pH (>6), Ashworth and Alloway [70] reported that Zn was released from biosolids and adsorbed by soil colloids. The highest Zn concentrations occurred in the bottom leaves at the end of the growing season, which is when willow may be increasingly fed to stock due to reduced pasture growth. 

The reduction in Cu, and to a lesser extent Zn, caused by biochar addition (both to the control and the biosolids treatments) may be due a pH increase, which reduces the solubility of these elements [71]. In addition, the biochar may provide both chemical and physical sorption sites for the elements [27,33]. Application of biochar to the soil was found to increase soil cation exchange capacity (CEC) by up to 40% and soil pH by up to one pH unit [72]. Since the negatively charged organic functional groups of the biochar increase over time during its oxidation in soil [25], and since the structure of biochar is highly recalcitrant in the soil environment to microbial decomposition [73], any biochar-trace element complexes formed are expected to be more stable than those formed with other forms of organic matter in soil [74]. Vacha et al. [75] reported a large variation in the capacity of various biochars to sorb trace elements, indicating that the performance of each system needs to be assessed individually.

The negligible change in plant Cd uptake in the biochar treatments is consistent with other authors reporting that liming may occasionally be ineffective for reducing plant Cd uptake [76,77]. This may be due to plants accumulating more Cd because there is less competition with Zn for transporter sites on the roots, or because of upregulation of Zn-transporter genes, which may also increase Cd uptake [78].

The foliar trace element concentrations in these experiments were a function of the Ca(NO_3_)_2_—extractable concentration in the soil, a finding that has been reported for *Lolium perenne* L. [58]. That the results were consistent both in the pot trial and in the field trial indicates that biosolids phytoremediation using willow will behave similarly on a large scale. This is not always the case; using *S. alba* X *S. viminalis*, Rosselli et al. [48] reported bioaccumulation coefficients of 0.95 for Zn and 1.42 for Cd in pot-grown samples but only 0.37 for Zn and 0.83 for Cd in field grown plants. 

While biosolids can accelerate the growth of willow and produce animal fodder that is biofortified in both Cu and Zn, it is unclear how long this effect will last. Assuming 10 t of shoots and foliar material is removed annually, the initial harvest would remove some 10 kg ha^−1^ of Zn from the soil. This is a significant fraction of the c.a. 44 kg of Zn added with the biosolids (assuming a biosolids addition rate of 50 t ha^−1^). The decrease in plant trace element uptake may be disproportionately large if there is an inordinate reduction in soluble Zn as has been reported [79]. In the case of Cd, the extraction was equivalent <12 g, which is small compared to the amount added (ca. 1.4 kg ha^−1^). Regarding Cu, the removal (<10 g ha^−1^) was negligible compared to the 12 kg ha^−1^ equiv. that was added. Therefore, if there were repeated applications of biosolids to maintain Zn uptake, not only would there be significant increases in soil Cd and Cu, but the Zn: Cd ratio would decrease. Both these factors would lead to increased Cd uptake and bioaccessibility [63,64]. Biosolids can increase dissolved organic matter, which can increase downward mobility of these contaminants, possibly into receiving waters [80].

## 5. Conclusions

Our results indicate that willows have potential for the phytoremediation of biosolids, where biosolids are applied to degraded or marginal land and the biomass. Except for Cd, the concentrations of other contaminants in the willows were not significantly increased. Our results supported the hypothesis that biochar reduces the bioavailability of some TEs in biosolids, at least in the case of willows, with the critical exception of Cd. This effect was most likely due to the high pH of the biochar. Willows grown on soils amended with biosolids or biosolids/biochar may produce Zn-biofortified stock fodder that may alleviate widespread Zn deficiencies, offset greenhouse gas emissions, and at the same time increasing biomass production since the mixture biosolids/biochar results in the highest biomass production. Critical next steps in this research are the determination of the bioaccessibility of willow-borne Cd to animals as well as assessing the change in trace element accumulation over several growing seasons.

## Figures and Tables

**Table 1:** Properties of the substrates used in the greenhouse experiments. The biosolids and biochar were added at 4% *w*/*w*. All values are in mg kg^−1^ (2 s.f.) unless otherwise stated. Values in brackets indicate the standard deviation of the mean (n = 5).

Pseudo-Total				
	Control	Biosolids	Biochar	Biosolids/Biochar
C %	3.3 (0.4)	3.7	5.6	5.8
N %	0.22 (0.01)	0.31	0.24	0.36
P	672 (21)	814 (24)	770 (8)	834 (12)
K	3209 (102)	3521 (64)	3565 (107)	3671 (29)
S	296 (8)	482 (22)	353 (5)	505 (13)
Ca	3058 (52)	3261 (52)	3664 (69)	3325 (46)
Mg	3341 (37)	3387 (9)	3227 (45)	3240 (14)
Na	206 (5)	219 (3)	240 (14)	215 (2)
As	3.3 (0.1)	3.7 (<0.1)	3.4 (0.1)	3.8 (0.1)
B	15 (1)	14 (1)	15 (<1)	15 (<1)
Cd	0.30 (0.01)	0.39 (0.02)	0.35 (0.01)	0.40 (0.02)
Cr	59 (11)	60 (1)	49 (9)	56 (10)
Cu	15 (3)	52 (11)	20 (6)	74 (1)
Fe	32,711 (5917)	30,809 (1014)	33,051 (5981)	43,327 (1900)
Mn	1188 (229)	1098 (49)	935 (175)	1123 (18)
Mo	0.55 (0.09)	0.90 (0.05)	0.47 (0.12)	1.11 (0.03)
Ni	30 (5)	29 (1)	23 (5)	30 (1)
Pb	47 (8)	53 (1)	38 (7)	52 (1)
Zn	59 (1)	90 (4)	62 (1)	98 (1)
Ca(NO_3_)_2_-extractable				
As	0.03 (<0.01)	0.02 (<0.01)	0.03 (0.01)	0.03 (<0.01)
Co	0.37 (0.01)	0.46 (<0.01)	0.23 (0.01)	0.35 (<0.01)
Cd	0.02 (<0.01)	0.05 (<0.01)	0.02 (<0.01)	0.04 (<0.01)
Cr	0.02 (<0.01)	0.02 (<0.01)	0.02 (<0.01)	0.02 (<0.01)
Cu	<0.01	0.12 (0.01)	0.02 (0.01)	0.13 (0.01)
Fe	8.8 (0.72)	10.8 (0.19)	5.4 (0.39)	7.9 (0.22)
Mn	46 (1)	53 (1)	47 (1)	44 (1)
Ni	0.15 (<0.01)	0.26 (0.01)	0.09 (<0.01)	<0.01
Zn	1.7 (0.08)	16 (1)	1.1 (0.05)	13 (1)

## Data Availability

Data will be made available on https://www.kiwiscience.com/journal-articles.html.

## Supplementary Materials

The following supporting information can be downloaded at: https://www.mdpi.com/article/10.3390/life13010243/s1, Table S1: Soil chemical properties of the biosolids and biochar. Values in brackets represent the standard error of the mean (n = 3 unless otherwise indicated). Adapted from Knowles et al. (2011) Gartler et al. (2013), and Taghizadeh-Toosi et al. (2011); Table S2: Elemental concentrations in the basal leaves (October harvest). All concentrations in mg/kg. Values in brackets represent the standard error of the mean (n = 5); Table S3: Elemental concentrations in the basal leaves (November harvest). All concentrations in mg/kg. Values in brackets represent the standard error of the mean (n = 5); Table S4: Elemental concentrations in the basal leaves (December harvest). All concentrations in mg/kg. Values in brackets represent the standard error of the mean (n = 5); Table S5: Elemental concentrations in the basal leaves (final harvest). All concentrations in mg/kg. Values in brackets represent the standard error of the mean (n = 5); Table S6: Elemental concentrations in the apical leaves (final harvest). All concentrations in mg/kg. Values in brackets represent the standard error of the mean (n = 5); Table S7: Elemental concentrations in the medial leaves (final harvest). All concentrations in mg/kg. Values in brackets represent the standard error of the mean (n = 5); Table S8: Elemental concentrations in the basal shoots (final harvest). All concentrations in mg/kg. Values in brackets represent the standard error of the mean (n = 5); Table S9: Elemental concentrations in the medial shoots (final harvest). All concentrations in mg/kg. Values in brackets represent the standard error of the mean (n = 5); Table S10: Elemental concentrations in the apical shoots (final harvest). All concentrations in mg/kg. Values in brackets represent the standard error of the mean (n = 5); Table S11: Elemental concentrations in the roots (final harvest). All concentrations in mg/kg. Values in brackets represent the standard error of the mean (n = 5).
