# Supplementary material for: Trace Element Uptake by Willows Used for the Phytoremediation of Biosolids
Source: Life (Basel). 2023 Jan 16;13(1):243. doi: 10.3390/life13010243 (PMC9862635; doi:10.3390/life13010243)
Supplement: Supplementary file 1 [file life-13-00243-s001.zip › life-2133998-supplementary.pdf]

Table S1. Soil chemical properties of the biosolids and biochar. Values in brackets represent the standard error of the mean (n=3 unless otherwise indicated). Adapted from Knowles et al, (2011) Gartler et al. (2013), and Taghizadeh-Toosi et al (2011).

	Soil	Biosolids	Biochar
pH	5.6 (5.6 – 5.7)	4.1	7.8
C (%)	3.3 (0.4)	28.0 (0.2)	70.6ǂ
N (%)	0.22 (0.01)	2.7 (0.03)	0.2ǂ
P	672 (21)	4683 (2)	412 (2)
K	3209 (102)	4330 (67)	1713 (17)
S	296 (8)	6972 (43)	288 (12)
Ca	3058 (52)	9818 (176)	7758 (160)
Mg	3341 (37)	2204 (17)	605 (11)
Na	206 (5)	428 (3)	1000 (29)
As	3.3 (0.1)	n.d.	n.d.
B	15 (1)	n.d.	n.d.
Cd	0.3 (0.01)	2.8 (0.0)	0.1 (0.01)
Cr	59 (11)	32 (1.4)	2.8 (0.6)
Cu	15 (3)	561(33)	14 (5)
Fe	32711 (5917)	n.d.	n.d.
Mn	1188 (229)	n.d.	n.d.
Mo	0.55 (0.09)	n.d.	n.d.
Ni	30 (5)	n.d.	n.d.
Pb	47 (8)	96 (3)	1.0 (0.2)
Zn	59 (1)	878 (13)	16 (1.3)

n.d. = "not determined"

ǂSingle analysis on homogenised material

Gartler, Jörg, Brett Robinson, Karen Burton, and Lynne Clucas. 2013. "Carbonaceous Soil Amendments to Biofortify Crop Plants with Zinc." *The Science of the Total Environment* 465 (November): 308–13.

Knowles, O. A., B. H. Robinson, A. Contangelo, and L. Clucas. 2011. "Biochar for the Mitigation of Nitrate Leaching from Soil Amended with Biosolids." *The Science of the Total Environment* 409 (17): 3206–10.

Taghizadeh-Toosi, Arezoo, Tim J. Clough, Leo M. Condron, Robert R. Sherlock, Craig R. Anderson, and Robin A. Craigie. 2011. "Biochar Incorporation into Pasture Soil Suppresses in Situ Nitrous Oxide Emissions from Ruminant Urine Patches." *Journal of Environmental Quality* 40 (2): 468–76.

Table S2: Elemental concentrations in the basal leaves (October harvest). All concentrations in mg/kg. Values in brackets represent the standard error of the mean (n=5).

	control		biosolids-biochar		biochar		biosolids	
Al	49.86	(3.32)	28.52	(7.45)	25.31	(1.95)	49.68	(2.72)
As			2.79	(2.34)				
B	68.94	(3.16)	61.21	(4.8)	56.98	(2.41)	66.15	(1.95)
Ca	17632.03	(956.02)	18006.93	(4336.99)	22204.50	(971.07)	23199.93	(617.71)
Cd	1.30	(0.04)	1.51	(0.3)	1.14	(0.11)	1.88	(0.1)
Co	0.47	(0.12)	0.67	(0.47)	0.15	(0.01)	0.21	(0.01)
Cr	0.81	(0.15)	1.51	(0.7)	0.46	(0.03)	0.46	(0.03)
Cu	5.17	(0.21)	9.27	(0.73)	5.46	(0.44)	10.46	(0.54)
Fe	54.97	(2.86)	44.43	(8.01)	44.45	(3.08)	52.90	(3.28)
K	16492.74	(584.25)	14370.55	(3825)	15048.56	(441.2)	18805.51	(1150.04)
Mg	4699.70	(261.79)	3364.28	(877.91)	3907.00	(230.13)	5171.38	(274.54)
Mn	160.71	(9.01)	97.14	(19.14)	102.96	(7.13)	185.82	(6.05)
Mo	0.06		1.46	(0.29)			0.87	(0.07)
Na	235.13	(22.1)	1042.65	(713.08)	297.89	(21.99)	355.89	(22.9)
Ni	0.70	(0.08)	1.66	(0.58)	0.36	(0.05)	0.96	(0.12)
P	2340.53	(139.52)	2109.72	(531.15)	2192.72	(49.04)	2622.53	(120)
Pb	0.15	(0.06)	2.30	(2.1)	0.30	(0.02)	0.16	(0.03)
S	2236.02	(241.56)	6380.62	(1471.4)	2841.03	(47.95)	8441.75	(797.44)
Zn	168.38	(14.31)	520.34	(36.3)	114.04	(7.56)	730.40	(47.72)

Table S3: Elemental concentrations in the basal leaves (November harvest). All concentrations in mg/kg. Values in brackets represent the standard error of the mean (n=5).

	control		biosolids-biochar		biochar		biosolids	
Al	37.50	(3.03)	35.36	(2.78)	29.93	(4.04)	40.07	(4.98)
As	0.60		0.60	(0.15)	0.43	(0.03)	0.96	(0.27)
B	63.94	(4.66)	61.10	(3.67)	52.83	(1.8)	53.50	(4.16)
Ca	18561.40	(1576.07)	23389.76	(977.08)	21839.32	(1080.92)	24299.22	(1145.13)
Cd	3.18	(0.27)	3.34	(0.21)	2.73	(0.27)	3.01	(0.15)
Co	0.24	(0.04)	0.14	(0.02)	0.23	(0.03)	0.22	(0.07)
Cr	0.43	(0.03)	0.49	(0.06)	0.58	(0.11)	0.65	(0.07)
Cu	9.78	(0.43)	19.56	(1.11)	11.42	(0.67)	22.21	(0.87)
Fe	90.23	(8.4)	89.41	(8.25)	90.12	(2.69)	85.57	(3.35)
K	63600.46	(5116.46)	73766.80	(3878.25)	67809.12	(3674.41)	78095.81	(3443.78)
Mg	5379.35	(454.44)	5778.07	(179.9)	5619.81	(296.28)	5676.30	(290.31)
Mn	169.87	(17.03)	161.71	(9.8)	158.36	(8.65)	178.53	(12.67)
Mo	0.24	(0.05)	0.88	(0.1)	0.26	(0.03)	0.62	(0.04)
Na	98.16	(8.26)	158.13	(14.57)	147.67	(27.55)	184.94	(17.54)
Ni	0.89	(0.08)	1.20	(0.16)	0.83	(0.11)	1.66	(0.24)
P	4191.67	(329.2)	4764.06	(209.58)	4704.63	(162.49)	5015.08	(233.06)
Pb							0.42	
S	4875.95	(612.02)	14785.52	(980.13)	7884.88	(224.24)	15389.57	(760.49)
Zn	158.02	(11.98)	451.76	(13.81)	145.13	(7.2)	600.57	(36.07)

Table S4: Elemental concentrations in the basal leaves (December harvest). All concentrations in mg/kg. Values in brackets represent the standard error of the mean (n=5).

	control		biosolids-biochar		biochar		biosolids	
Al	96.56	(18.12)	84.95	(13.25)	62.43	(13.2)	102.75	(15.73)
As	0.21	(0.08)	0.39	(0.05)	0.43	(0.05)	0.48	(0.08)
B	111.41	(11.85)	104.93	(5.81)	107.01	(3.12)	116.46	(10.67)
Ca	20388.31	(1745.45)	29472.72	(2563.37)	28751.19	(2745.93)	29980.84	(1372.96)
Cd	1.76	(0.14)	3.16	(0.24)	1.79	(0.23)	2.94	(0.16)
Co	0.35	(0.02)	0.15	(0.02)	0.29	(0.08)	0.36	(0.05)
Cr	0.78	(0.13)	1.07	(0.27)	0.84	(0.1)	0.92	(0.12)
Cu	4.94	(0.49)	9.49	(1.49)	5.21	(0.33)	10.06	(0.43)
Fe	97.79	(21.45)	101.07	(17.77)	78.71	(8.8)	97.58	(20.77)
K	13683.66	(1242.92)	13375.16	(916.44)	11100.49	(1063.56)	12752.38	(748.34)
Mg	5410.35	(450.59)	5599.35	(470.8)	4861.71	(553.96)	6674.34	(398.89)
Mn	206.74	(16.55)	181.01	(11.89)	146.68	(15.82)	313.87	(20.94)
Mo	0.26	(0.05)	1.44	(0.15)	0.26	(0.02)	1.25	(0.11)
Na	247.49	(31.39)	369.11	(40.55)	328.27	(32.35)	396.81	(23.24)
Ni	0.73	(0.07)	1.37	(0.11)	0.74	(0.11)	1.71	(0.22)
P	1802.87	(136.44)	3010.95	(739.33)	1725.46	(70.88)	2900.67	(170.23)
Pb	0.44	(0.04)	0.46	(0.09)	0.41	(0.07)	0.38	(0.09)
S	2343.17	(143.16)	8785.25	(1402.69)	3188.91	(253.29)	10448.80	(936.97)
Zn	271.42	(20.6)	925.42	(66.66)	211.06	(17.38)	1185.59	(96.1)

Table S5: Elemental concentrations in the basal leaves (final harvest). All concentrations in mg/kg. Values in brackets represent the standard error of the mean (n=5).

$mg*kg^{-1}$	control		biosolids-biochar		biochar		biosolids	
Al	111.35	(19.3)	120.18	(14.4)	114.32	(12.33)	140.03	(18.2)
As	0.48	(0.12)	0.35	(0.05)	0.30	(0.03)	0.77	
B	99.93	(7.25)	92.54	(5.84)	95.24	(4.55)	101.00	(2.8)
Ca	19692.38	(2027.57)	23680.85	(1086.48)	24580.81	(635.16)	24956.23	(1052.68)
Cd	1.76	(0.16)	2.59	(0.12)	1.49	(0.09)	2.43	(0.14)
Co	0.37	(0.04)	0.16	(0.06)	0.15	(0.01)	0.25	(0.02)
Cr	1.95	(0.3)	2.62	(0.53)	2.68	(0.28)	2.24	(0.48)
Cu	4.84	(0.47)	9.16	(1.23)	5.04	(0.22)	10.04	(0.63)
Fe	122.98	(18.51)	151.15	(17.42)	151.21	(17.33)	152.64	(19.25)
K	14921.74	(698.95)	17151.02	(1202.03)	14160.19	(456.3)	16445.85	(774.71)
Li	0.24	(0.04)	0.40	(0.14)	0.42	(0.1)		
Mg	4803.65	(430.08)	4651.88	(142.92)	4138.65	(202.3)	5751.21	(157.08)
Mn	209.79	(18.59)	162.68	(8.98)	128.93	(7.45)	290.50	(13.86)
Mo	0.24	(0.03)	1.44	(0.14)	0.25	(0.01)	1.40	(0.15)
Na	200.42	(25.47)	271.18	(22.59)	262.41	(22.5)	286.56	(11.97)
Ni	0.98	(0.12)	1.80	(0.25)	1.15	(0.08)	2.13	(0.56)
P	1722.55	(152.29)	2968.27	(565.18)	1656.55	(123.13)	2960.78	(175.21)
Pb	0.35	(0.03)	0.36	(0.1)	0.26	(0.05)	0.30	(0.06)
S	2031.32	(177.9)	7200.44	(751.52)	2645.08	(140.42)	8738.60	(679.77)
Zn	267.99	(21.71)	812.24	(40.51)	206.12	(10.92)	1068.75	(71.95)

Table S6: Elemental concentrations in the apical leaves (final harvest). All concentrations in mg/kg. Values in brackets represent the standard error of the mean (n=5).

$mg*kg^{-1}$	control		biosolids-biochar		biochar		biosolids	
Al	60.99	(8.79)	70.52	(7.83)	65.77	(5.45)	61.21	(4.46)
As	0.17		0.35	(0.14)	0.24	(0.03)	0.32	(0.04)
B	85.16	(7.83)	88.38	(5.56)	87.41	(11.4)	75.73	(9.76)
Ca	9154.56	(964.23)	11781.52	(1027.5)	11597.59	(856.05)	9551.65	(1439.33)
Cd	1.58	(0.18)	1.37	(0.13)	1.50	(0.15)	1.15	(0.11)
Co	0.39	(0.05)	0.07	(0.01)	0.14	(0.02)	0.24	(0.04)
Cr	1.22	(0.07)	1.40	(0.2)	1.57	(0.22)	1.10	(0.12)
Cu	7.64	(1.76)	12.49	(0.96)	7.83	(0.79)	16.43	(2.6)
Fe	106.48	(9.84)	120.50	(13.16)	115.53	(6.49)	106.32	(2.33)
K	22177.46	(6164.94)	19376.28	(1995.26)	17364.45	(937.81)	22590.03	(1630.64)
Li	0.09	(0.01)	0.11	(0.02)	0.12	(0.02)		
Mg	3511.67	(191.02)	3540.52	(104.2)	3232.94	(182.61)	3516.90	(277.42)
Mn	109.91	(12.11)	85.99	(11.4)	60.12	(5.34)	123.12	(16.67)
Mo	0.20	(0.02)	0.76	(0.06)	0.20	(0.02)	0.80	(0.14)
Na	88.87	(7.23)	109.13	(8.29)	109.99	(12.06)	105.39	(15.16)
Ni	1.88	(0.49)	2.34	(0.34)	1.59	(0.14)	3.38	(0.75)
P	2600.99	(665.27)	2851.61	(204.35)	2182.00	(326.14)	3662.91	(417.35)
Pb	0.16	(0.06)	0.36	(0.01)	0.41	(0.14)	0.21	(0.03)
S	2855.19	(319.06)	5147.40	(148.57)	3022.64	(113.76)	5701.31	(375.33)
Zn	145.78	(17.23)	348.58	(30.99)	119.46	(10.08)	376.92	(76.05)

Table S7: Elemental concentrations in the medial leaves (final harvest). All concentrations in mg/kg. Values in brackets represent the standard error of the mean (n=5).

mg*kg^{-1}	control		biosolids-biochar		biochar		biosolids	
Al	127.47	(18.29)	113.88	(10.8)	113.66	(5.09)	114.26	(13.26)
As	0.25	(0.04)	0.41	(0.07)	0.37	(0.03)	0.37	(0.1)
B	97.12	(2.4)	121.33	(5.51)	93.63	(24.59)	93.36	(3.3)
Ca	16652.71	(713.13)	18575.02	(1170.85)	19619.91	(796.14)	20279.20	(510.43)
Cd	1.79	(0.14)	2.09	(0.1)	1.08	(0.28)	2.05	(0.1)
Co	0.32	(0.02)	0.07	(0.01)	0.12	(0.01)	0.23	(0.02)
Cr	2.28	(0.46)	2.50	(0.44)	2.00	(0.3)	1.83	(0.33)
Cu	4.94	(0.4)	9.12	(0.85)	5.71	(0.26)	10.68	(0.76)
Fe	138.16	(18.26)	152.11	(15.16)	166.94	(8.82)	136.59	(12.02)
K	15039.95	(506.38)	17154.72	(959.72)	14856.36	(934.64)	15148.22	(1121.28)
Li	0.15	(0.01)	0.29	(0.07)	0.25	(0.03)		
Mg	4472.46	(193.44)	4145.45	(154.96)	3578.64	(102.78)	5137.54	(246.17)
Mn	195.78	(9.52)	136.65	(4.86)	114.42	(6.15)	261.58	(13.59)
Mo	0.21	(0.02)	1.29	(0.16)	0.31	(0.04)	1.25	(0.15)
Na	149.72	(8.52)	208.09	(17.02)	203.55	(9.7)	199.66	(7.09)
Ni	0.94	(0.13)	1.75	(0.17)	1.05	(0.13)	1.85	(0.27)
P	1811.02	(152.58)	2801.21	(337.59)	1676.64	(38.09)	2968.35	(141.31)
Pb	0.33	(0.05)	0.47	(0.09)	0.35	(0.1)	0.33	(0.12)
S	2118.23	(130.37)	6492.21	(624.63)	2485.02	(121.65)	7196.91	(548.21)
Zn	262.28	(15.55)	686.61	(65.56)	161.06	(42.21)	859.30	(43.47)

Table S8: Elemental concentrations in the basal shoots (final harvest). All concentrations in mg/kg. Values in brackets represent the standard error of the mean (n=5).

$mg*kg^{-1}$	control		biosolids-biochar		biochar		biosolids	
Al	83.66	(6.53)	101.69	(8.19)	102.53	(9.11)	99.20	(8.88)
As					0.21	(0.08)	0.23	(0.01)
B	17.06	(1.89)	15.64	(1.08)	18.23	(1.39)	15.84	(0.81)
Ca	7640.49	(455.48)	9100.80	(624.75)	9917.15	(356.53)	8927.04	(342.23)
Cd	2.43	(0.16)	2.38	(0.13)	2.29	(0.11)	2.12	(0.12)
Co	0.07	(0.01)			0.04	(0.01)	0.09	(0.03)
Cr	1.48	(0.19)	1.74	(0.17)	1.41	(0.15)	1.74	(0.14)
Cu	3.74	(0.35)	8.77	(0.46)	3.82	(0.19)	12.49	(0.46)
Fe	61.86	(9.43)	64.26	(4.79)	64.26	(6.21)	63.83	(4.43)
K	4913.24	(390.14)	4905.23	(684.16)	5804.44	(229.73)	6198.79	(151.88)
Li	0.11		0.12		0.12			
Mg	920.69	(55.55)	934.64	(54.03)	886.68	(58.88)	1157.97	(39.87)
Mn	84.57	(11.58)	63.65	(3.45)	60.20	(4.52)	105.04	(5.72)
Mo	0.09	(0.02)	0.14	(0.02)	0.14	(0.05)	0.14	(0.01)
Na	390.96	(61.62)	471.54	(31.32)	480.62	(24.38)	490.71	(9.53)
Ni	0.68	(0.08)	0.98	(0.17)	0.63	(0.07)	2.20	(1.01)
P	1095.98	(54.91)	1390.78	(19.59)	1124.72	(28.24)	1517.36	(57.65)
Pb	0.41	(0.2)	0.25	(0.01)	0.35	(0.04)	0.23	(0.03)
S	629.59	(27.49)	1054.54	(85.49)	777.38	(55.04)	1190.98	(54.33)
Zn	145.33	(7.82)	293.45	(19.19)	126.71	(5.72)	348.18	(22.57)

Table S9: Elemental concentrations in the medial shoots (final harvest). All concentrations in mg/kg. Values in brackets represent the standard error of the mean (n=5).

$mg*kg^{-1}$	control		biosolids-biochar		biochar		biosolids	
Al	44.84	(1.45)	42.67	(2.74)	44.59	(2.12)	41.59	(2.31)
As	0.16	(0.03)	0.37		0.20	(0.05)	0.19	(0.03)
B	13.76	(0.49)	12.40	(0.3)	13.81	(0.27)	12.86	(0.6)
Ca	5369.16	(173.13)	6323.68	(245.6)	7599.00	(708.67)	5641.73	(303.05)
Cd	2.01	(0.03)	1.78	(0.03)	1.85	(0.11)	1.48	(0.09)
Co	0.07		0.03	(0.01)	0.03		0.05	
Cr	0.75	(0.11)	0.73	(0.07)	0.76	(0.04)	0.73	(0.16)
Cu	3.31	(0.13)	8.38	(0.27)	3.84	(0.23)	10.50	(0.37)
Fe	26.14	(1.66)	25.60	(2.75)	28.49	(1.92)	24.35	(1.86)
K	5808.78	(101.51)	5299.18	(473.79)	5937.23	(258.17)	6260.41	(113.34)
Li	0.10		0.11	(0.01)	0.12	(0.01)		
Mg	825.29	(32.28)	810.12	(62.43)	768.61	(65.07)	964.30	(38.36)
Mn	56.98	(0.93)	36.44	(2.37)	32.90	(1.79)	62.29	(4.21)
Mo	0.05	(0.01)	0.12	(0.01)	0.08	(0.02)	0.12	(0.02)
Na	295.68	(17.05)	345.79	(17.19)	385.32	(30.59)	304.83	(10.25)
Ni	0.44	(0.14)	0.63	(0.15)	0.49	(0.06)	0.47	(0.12)
P	1200.15	(29.4)	1568.59	(67.27)	1239.87	(19.86)	1549.11	(58.62)
Pb	0.18	(0.06)	0.30	(0.04)	0.32	(0.02)	0.33	(0.03)
S	723.35	(3.57)	1181.03	(41.41)	841.27	(27.93)	1254.22	(59.99)
Zn	105.93	(5.05)	229.59	(4.08)	87.15	(7.14)	253.96	(23)

Table S10: Elemental concentrations in the apical shoots (final harvest). All concentrations in mg/kg. Values in brackets represent the standard error of the mean (n=5).

$mg*kg^{-1}$	control		biosolids-biochar		biochar		biosolids	
Al	50.18	(3.91)	47.49	(3.32)	48.47	(1.65)	52.68	(3.15)
As	0.00	(0)	0.11	(0)	0.17	#DIV/0!	0.62	(0.41)
B	25.38	(3.11)	20.94	(1.62)	24.00	(1.65)	28.47	(2.49)
Ca	5487.46	(436.35)	6401.78	(410.83)	6411.98	(645.39)	5464.03	(425.65)
Cd	2.45	(0.16)	1.99	(0.13)	2.18	(0.23)	1.72	(0.12)
Co	0.09	(0.01)	0.05	(0.01)	0.10	(0.04)	0.09	(0.02)
Cr	0.95	(0.22)	0.87	(0.07)	0.72	(0.09)	0.81	(0.07)
Cu	4.65	(0.27)	9.87	(0.48)	4.80	(0.66)	13.41	(1.1)
Fe	38.35	(7.33)	34.67	(4.08)	32.59	(2.14)	39.62	(3.83)
K	11264.24	(1195.07)	12291.24	(1116.43)	14707.45	(1636.11)	16900.30	(2198.87)
Li	0.12	(0.02)	0.12	(0.01)	0.12	(0.01)		
Mg	1168.81	(53.12)	1259.31	(171.46)	1036.40	(5.51)	1656.45	(285.58)
Mn	64.10	(8.73)	42.77	(4.72)	39.03	(5.44)	67.29	(9.4)
Mo	0.27	(0.09)	0.29	(0.06)	0.15	(0.03)	0.33	(0.02)
Na	465.55	(74.26)	418.95	(48.28)	476.98	(69.2)	424.17	(26.49)
Ni	1.29	(0.61)	1.05	(0.36)	0.72	(0.19)	1.46	(0.15)
P	1801.26	(69.8)	2370.40	(167.39)	1997.32	(262.16)	2913.85	(114.11)
Pb	0.35	(0.04)	0.61	(0.05)	0.53	(0.07)	0.72	(0.23)
S	1609.27	(91.58)	2789.42	(345.83)	1800.87	(243.01)	3505.61	(201.45)
Zn	118.57	(16.95)	256.64	(29.51)	98.48	(15.25)	274.66	(36.73)

Table S11: Elemental concentrations in the roots (final harvest). All concentrations in mg/kg. Values in brackets represent the standard error of the mean (n=5).

$mg*kg^{-1}$	control		biosolids-biochar		biochar		biosolids	
Al	1057.67	(127.32)	934.35	(109.99)	556.04	(71.54)	1316.91	(234.17)
As	0.43	(0.09)	0.60	(0.14)	0.30	(0.05)	0.90	(0.2)
B	10.58	(0.29)	9.80	(0.33)	10.27	(0.52)	9.76	(0.67)
Ca	6743.39	(343.84)	6203.10	(196.62)	7192.43	(156.73)	5561.70	(222.91)
Cd	0.70	(0.05)	0.94	(0.04)	0.65	(0.05)	1.01	(0.11)
Co	0.33	(0.06)	0.23	(0.03)	0.18	(0.03)	0.34	(0.08)
Cr	5.06	(0.86)	7.14	(0.64)	2.80	(0.34)	11.73	(3.51)
Cu	6.51	(0.36)	23.11	(1.45)	7.31	(0.36)	40.74	(9.15)
Fe	716.72	(93.55)	632.39	(56.79)	431.34	(54.92)	1479.10	(536.64)
K	8513.74	(647.1)	7803.31	(692.95)	8777.30	(380.74)	8193.41	(869.03)
Li	0.18	(0.06)	0.12	(0.07)	0.05	(0.01)		
Mg	1491.13	(116.36)	1380.64	(61.68)	1396.14	(96.95)	1488.04	(95.36)
Mn	84.12	(10.87)	75.13	(6.52)	50.42	(3.24)	149.46	(39.89)
Mo	0.19	(0.05)	2.07	(0.11)	0.22	(0.03)	2.68	(0.6)
Na	691.14	(89.86)	755.11	(54.06)	708.80	(83.22)	592.20	(83.24)
Ni	2.93	(0.31)	4.41	(0.38)	2.04	(0.23)	6.91	(1.51)
P	2492.37	(77.9)	2904.51	(102.77)	2579.76	(106.29)	3072.89	(169.96)
Pb	0.91	(0.14)	1.52	(0.16)	0.48	(0.09)	3.42	(1.21)
S	1700.19	(176.72)	2858.76	(168.66)	1834.21	(118)	3259.28	(451.51)
Zn	73.13	(4.61)	213.27	(6.6)	60.86	(4)	282.45	(34.81)
